# Comparison of Electrocardiogram and QT Interval between Viral Hepatitis Cirrhosis and Alcoholic Cirrhosis

**DOI:** 10.1155/2022/6934418

**Published:** 2022-10-18

**Authors:** Li-Hong Lu, Xue-Ya Lv, Qi-Ming Wu, Qian Dong, Zhao Wang, Su-Juan Zhang, Li Fu, Qian Wang, Yu-Qing Song

**Affiliations:** Department of Cardiology, Beijing Ditan Hospital, Capital Medical University, Beijing 100015, China

## Abstract

**Objective:**

This study aims to compare the electrocardiogram (ECG) abnormalities and QT interval prolongation in 2,886 patients with viral hepatitis cirrhosis and 643 patients with alcoholic cirrhosis in order to understand the characteristics of ECG in patients with cirrhosis and provide information and evidence for clinical diagnosis and treatment.

**Methods:**

The ECG data of patients with viral hepatitis cirrhosis and alcoholic liver cirrhosis in the outpatients and inpatients of our hospital from August 2012 to July 2018 were reviewed. The ECG data were recorded, and the ECG report was issued by ECG experts to analyze the abnormal ECG and QT interval of patients in these two groups.

**Results:**

In the present study, 1,132 (39.22%) of the 2,886 patients with viral liver cirrhosis and 322 (50.08%) of the 643 patients with alcoholic liver cirrhosis had an abnormal ECG (*P* < 0.001). Among patients with QT prolongation, 388 patients had viral liver cirrhosis (13.44%) and 170 patients had alcoholic liver cirrhosis (26.44%, *P* < 0.001).

**Conclusion:**

The hemodynamics and electrophysiology of the myocardium are often changed in patients with cirrhosis, and ECG changes may also occur. QT interval prolongation is one of the most common electrophysiological abnormalities in patients with cirrhosis, and QT prolongation is more common in patients with alcoholic liver cirrhosis. Prolonged QT is associated with severe arrhythmia and sudden death and can warn of malignant arrhythmia and sudden death. Therefore, the routine detection of abnormal ECG and QT interval in patients with liver cirrhosis is of significant importance for preventing malignant events.

## 1. Introduction

Cirrhosis of the liver may affect cardiac functions and cause electrophysiological abnormalities of the heart. The electrophysiological abnormalities of the heart in cirrhosis include repolarization abnormality and chronotropic insufficiency. These changes would cause corresponding electrocardiogram (ECG) changes, which are the early manifestations of cardiac involvement in patients with cirrhosis. Therefore, improving the understanding of cardiac electrophysiology and ECG changes in patients with cirrhosis can provide early warning for cardiac diseases [1]. QT interval refers to the process from the beginning of the QRS wave to the end of T wave in ECG, which includes the depolarization and repolarization of ventricular muscles. QT interval prolongation is one of the most common electrophysiological abnormalities and one of the most fatal ECG changes in patients with cirrhosis [2]. This allows severe arrhythmias to be predicted early [2]. QT interval prolongation was first observed in patients with alcoholic liver disease [3]. QT interval prolongation is the most common electrophysiological change in CCM, occurring in more than 50% of cases, and can lead to severe arrhythmias, including ventricular arrhythmias and sudden cardiac death. Subsequently, QT prolongation was found to be common in patients with cirrhosis of all causes [4, 5]. Furthermore, QT interval prolongation is most common in patients with cirrhosis, and the incidence increases with the progression of the disease [6].

This study compared 2,886 cases of viral hepatitis cirrhosis and 643 cases of alcoholic cirrhosis with abnormal ECGs and prolonged QT intervals in order to explore the changes in ECG in patients with cirrhosis and provide information and evidence for clinical diagnosis and treatment.

## 2. Methods

### 2.1. General Data

The ECG data of the outpatient and inpatient patients with cirrhosis from August 2012 to July 2018 in our hospital were reviewed. All these cases met the diagnostic criteria of the viral hepatitis prevention and treatment program revised jointly by the Infectious Diseases and Parasitology Branch and Hepatology Branch of the Chinese Medical Association in 2000 [7]. Electrocardiogram of patients with clearly diagnosed viral hepatitis cirrhosis and alcoholic cirrhosis was selected for inductive analysis, while patients with cirrhosis caused by other causes were excluded.

This study was performed in accordance with the principles stated in the Declaration of Helsinki. Ethical approval was obtained from the Ethics Committee of Beijing Ditan Hospital, Capital Medical University. Due to the retrospective nature of the study, informed consent was waived.

### 2.2. Instruments and Methods

The 12-lead ECG was detected using the MAC5500 photoelectric ECG machine (GE, USA), and 12-lead ECG electrodes were placed in accordance with international unified standards. QT was automatically measured by the computer and was corrected, and ECG reports were issued by ECG experts. Reference range for QT interval is <420 ms for male and <440 ms for female. The formula for calculating QT interval is QT_C_ = QT/RR^1/2^.

### 2.3. Statistical Analysis

The SPSS 18.0 software package was used for the statistical analysis of the obtained data. The measurement data were expressed as mean ± standard deviation. Categorical data were described as numbers and percentages and compared using the chi-square test. A *P-*value of <0.05 was considered statistically significant.

## 3. Results

### 3.1. Complications of Liver Cirrhosis in the Two Groups

As summarized in Table 1, among these patients, there were 2,886 cases of viral hepatitis cirrhosis, which included 2,074 males and 812 females, and their age ranged within 18-78 years, with an average age of 55.37 ± 11.32 years. Furthermore, there were 643 cases of alcoholic cirrhosis, which included 634 males and nine females, and their age ranged within 26-86 years, with an average age of 53.14 ± 9.38 years. Among the 2,886 cases of viral hepatitis cirrhosis, 2,419 cases had hepatitis B cirrhosis, which accounted for the vast majority (83.82%) of this group, followed by hepatitis C cirrhosis (13.86%) and hepatitis D cirrhosis (2.32%). Among the 643 cases of alcoholic cirrhosis, diabetes was the most common complication in this group (8.09%).

### 3.2. Abnormal ECG of Patients with Cirrhosis

As is shown in Table 2, in the present study, 1,132 (39.22%) of the 2,886 patients with viral liver cirrhosis and 322 (50.08%) of the 643 patients with alcoholic liver cirrhosis had abnormal ECGs. The difference between these two groups was statistically significant (*P* < 0.001). Among patients with QT prolongation, there were 388 cases with viral liver cirrhosis (accounted for 13.44% of all viral liver cirrhosis patients) and 170 cases with alcoholic liver cirrhosis (accounted for 126.44% of all alcoholic liver cirrhosis patients). The difference in abnormal rate of ST segment and abnormal T wave between these two groups was statistically significant (*P* < 0.001).

### 3.3. Correlation between Child Pugh Score and QT Prolongation

QTc interval was positively correlated with Child-Pugh score (*r* = 0.38, *P* < 0.01). Child-Pugh score was an independent correlated factor affecting QTc interval in patients with cirrhosis (*t* = 2.50, *P* = 0.015), indicating that Child-Pugh score was an independent predictor of QTc interval length variability.

## 4. Discussion

There are different reports on the incidence of QT prolongation in patients with cirrhosis [8–14]. Li et al. [8] reported that 80% of patients with cirrhosis had various ECG abnormalities and that the QT interval was prolonged by up to 60%. Bal and Thuluvath [10] reported that 30%-50% patients with cirrhosis had QTc prolongation in ECG. Tahata et al. found that QTc prolongation was developed in 6.4% of Japanese patients during combination treatment of ledipasvir and sofosbuvir [11]. Zambruni et al. found that chronic beta-blockade shortens QT interval in liver cirrhosis patients with prolonged baseline values [12].

The possible mechanism of QT prolongation in patients with liver cirrhosis includes the following: (1) autonomic nerve dysfunction. Bal and Thuluvath [10] reported that the impaired autonomic nerve function of patients with cirrhosis, especially the dominant sympathetic nerve, formed the basis for the prolonged QTc interphase of end-stage liver disease. After liver transplantation, the autonomic nerve function improved, and the prolonged QTc interphase could recover to some extent. (2) Disturbance in sex hormone metabolism: Androgen can protect the electrophysiological stability of the heart. However, the relative lack of androgen in patients with liver cirrhosis and the prolongation of the QT interval may be correlated to the relative lack of androgen. (3) Liver function incomplete spontaneous portal shunting leads to the increase of some toxic metabolites, which can directly damage the myocardium, affect the myocardial repolarization, and lead to a prolonged QT interval [8].

The present study revealed that there were significant differences in QT prolongation between the two groups and that patients with QT prolongation in the alcoholic cirrhosis group were significantly greater than those in the hepatitis cirrhosis group. There are more patients with QT prolongation in the alcoholic cirrhosis group. The possible reason is that the changes in QT interval and action potential duration (APD) are correlated to the function of the ion channel of cardiac muscle cells. Existing studies have proven that the cardiac muscle cells of various animals can affect APD under the effect of alcohol, thereby prolonging the QT interval. It was reported that five minutes of alcohol exposure downregulated the APD of Purkinje fibers in the heart of dogs. Hypokalemia weakened K+ outward repolarization current, thus delayed repolarization and prolonged QT. Fluoroquinolone has good antibacterial activities against Gram-negative bacteria, Gram-positive bacteria, mycoplasma, and chlamydia, with few adverse reactions and good pharmacokinetics. They can inhibit the activity of bacterial DNA rotase, thereby preventing the synthesis and replication of bacterial DNA, and play an anti-infection role. Another study showed that Rhizoma Dioscoreae bulbiferae could cause hepatotoxicity *via* oxidative damage in mice, and mitochondria might be the targets [15].

Jiang et al. [16] reported that after five months of alcohol feeding and intragastric administration, pathological examination revealed that the myofilaments of mouse myocardial cells were irregularly broken, and that these myofilaments were broken under an electron microscope, the gap junction area of the run disk was broken, the dense spots disappeared, and lipid droplets were formed. Hence, the ECG QTc was significantly prolonged. These characteristics are consistent with those in relevant reports [17–19]. In the whole-cell patch-clamp experiment, the APD90 of alcohol-fed mice was prolonged, the inactivation of calcium channels was delayed, and the window current of calcium channels was increased, suggesting that the function of calcium channels was upregulated. The upregulation in calcium channel function is one of the reasons for the QTc prolongation in patients with type 8 long QT syndrome. Boczek et al. [20] also reported that the mutated calcium channels opened earlier, the function was upregulated, the window current increased, and more calcium ions entered the cells, leading to the increase in potassium ions needed for repolarization. This is an important cause of abnormal ventricular repolarization, with the latter being the cardiac electrocardiography matrix of sudden death [21,22].

The QTc prolongation of the electrocardiogram is of important clinical significance for evaluating the condition and prognosis of patients with cirrhosis. QTc prolongation is correlated to severe arrhythmia and sudden death, and QTc prolongation is one of the common phenomena in patients with sudden death of alcoholic heart disease [23–25]. The probability of QTc prolongation in these patients is 22%-46.9% [4].

In addition, there are some limitations in the present study. This study was a retrospective study, without collecting the treatment and medication status of patients. Also, patients were not followed up. In the future, we will comprehensively consider the abovementioned issues and further improve the research.

Cirrhosis is a common disease with high incidence and poor prognosis. Patients often have changes in hemodynamics and cardiac electrophysiology and related ECG changes. As a noninvasive, rapid, and economical examination method, ECG can easily to be accepted by patients. Therefore, the routine ECG examination of patients with cirrhosis in clinical practice could provide an early warning of malignant arrhythmia and sudden death, thereby preventing the occurrence of malignant events. This has important guiding significance for clinical diagnosis and treatment [25].

## Figures and Tables

**Table 1:** Complications and proportion of cirrhosis in two groups [cases (%)].

Complication	Viral hepatitis cirrhosis (*n* = 2886)	Alcoholic cirrhosis (*n* = 643)	*P-*values
Hepatitis B	2419 (83.82)	32 (4.98)	<0.001
Hepatitis C	371 (13.86)	1 (0.16)	<0.001
B with hepatitis C	11 (0.4)	0 (0.00)	0.233
Other viral hepatitis	85 (2.9)	0 (0.00)	<0.001
Malignant tumor	266 (9.22)	14 (2.18)	<0.001
Gallbladder stones	38 (1.32)	2 (0.31)	0.061
Diabetes	192 (6.65)	52 (8.09)	0.195
High blood pressure	109 (3.78)	17 (2.64)	0.161
Coronary heart disease	14 (0.55)	2 (0.31)	0.752
HIV/AIDS	8 (0.23)	2 (0.31)	0.884

**Table 2:** Abnormal electrocardiogram and its composition proportion of patients with cirrhosis in two groups [cases (%)].

ECG	Hepatitis cirrhosis	Alcoholic cirrhosis
Total number	2886	643
Male	2074 (71.86)	634 (98.60)
Female	812 (28.14)	9 (1.40)
Age	55.37 ± 11.32	53.14 ± 9.38
Normal ECG	1712 (59.32)	309 (48.06)
Abnormal ECG	1132 (39.22)	322 (50.08)
QT interphase	388 (13.44)	170 (26.44)

*Note.* Comparison of QT interval between viral liver cirrhosis and alcoholic liver cirrhosis: *χ*^2^ = 66.70, *P* < 0.001.

## Data Availability

The data that support the findings of this study are available from the corresponding author upon reasonable request.
